# An hourglass model for conceptualising stigma in infectious disease outbreaks

**DOI:** 10.1038/s41598-025-98995-w

**Published:** 2025-05-02

**Authors:** Amy Paterson, Benjamin Jones, Olive Kabajaasi, Ashleigh Cheyne, Harun Tulunay, Kkunsa Hadson, Jeni Stolow, Nina Gobat, Piero Olliaro, Amanda Rojek

**Affiliations:** 1 Pandemic Sciences Institute, University of Oxford, Old Road Campus, Roosevelt Drive, Oxford, OX3 7DQ UK; 2 Nuffield Department of Medicine, Health Systems Collaborative, Oxford, UK; 3 Menzies School of Health Research, Darwin, Australia; 4 Walimu, Yuri Gagarin Rd, Kampala, Uganda; 5 Positively UK, 14 St. Marys House, Chillingworth Road, London, N7 8SH UK; 6 Medical and Molecular Laboratories, Kyanja, Kampala, Uganda; 7 Tulane University School of Public Health and Tropical Medicine, New Orleans, LA USA; 8 Global Outbreak Alert and Response Network, World Health Organization, Geneva, Switzerland; 9 Nuffield Department of Primary Care Health Services, University of Oxford, 32 Woodstock Road, Oxford, OX2 6HT UK

**Keywords:** Conceptual model, Discrimination, Emerging infectious diseases, Outbreak, Pandemic preparedness, Stigma, Infectious diseases, Health policy, Public health, Human behaviour

## Abstract

Stigma is widely observed during (re)emerging infectious disease outbreaks, contributing to psychological distress, social isolation, and care-seeking hesitancy. Despite this, it is often inadequately addressed in public health responses, partly due to the lack of a fit-for-purpose approach. The objective of this study was to develop a conceptual model to facilitate structured consideration of stigma during (re)emerging disease outbreaks. We conducted 34 in-depth interviews with international stakeholders across 25 outbreak-prone diseases, including emergency response leaders, frontline responders, researchers, and community advocates. We analysed transcripts using thematic analysis, integrating insights from social and behavioural theories to refine the model. We introduce the hourglass stigma model, a theory-informed conceptualisation of stigma in outbreaks. The model consists of five domains (major themes): context, thoughts, emotions, manifestations, and impact. Within each domain there are key considerations, such as the influence of response measures on concealability (context), the association of certain diseases with ‘dirtiness’ due to hygiene-dominant messaging (thoughts), the negative effects of fear-based appeals (emotions), the enactment of stigma due to unconscious bias (manifestations), and the enduring consequences of (mis)trust in institutions (impact). The hourglass model can be used to inform operational tools, ensuring stigma is adequately addressed in outbreak preparedness and response activities.

## Introduction

Stigma shapes the trajectory of new and re-emerging infectious disease outbreaks^1,2^. Its influence spans across most facets of outbreak control, from impeding timely health-seeking behaviours to diminishing uptake of preventive measures^3^. Stigma also affects community wellbeing, with adverse psychological and social ramifications for those with the disease, and those associated with it^2^. These impacts often linger, as seen in the aftermath of the 2014–2016 West Africa Ebola outbreak, where stigma hindered the reintegration of survivors into their communities^4^.

Globally, there is a shift in the approach to health emergency management. The WHO revised framework for Health Emergency Preparedness Response and Resilience emphasises the necessity of person- and community-centred emergency management^5^. This change is driven by a history of insufficient attention to social dynamics in outbreak responses, which can inadvertently cause harm by reinforcing existing inequities that contribute to stigmatisation^5^.

Despite its impact, stigma is poorly understood in outbreak contexts, with attempts at assessment typically disjointed and delayed^6^. As a result, stigma reduction interventions are limited in their theoretical underpinning, and occur in a haphazard manner, if at all^2,6^. This lack of a systematic approach to understanding and addressing stigma represents a critical gap in pandemic preparedness and emerging outbreak response. 

Stigma is defined as disapproval and discrimination due to an attribute or association deemed socially discrediting^7^. While models exist for understanding stigma associated with established diseases such as HIV^8^, tuberculosis^9^, and mental health conditions^10^, and health more broadly^11^, the unique features of outbreak-associated stigma require a tailored approach. These features, including heightened fear, transmission between people, infectious status disclosure obligations, and a lack of available information about disease transmission and severity, underscore the need for a specialised model to guide assessment and interventions.

Here, we develop a model to understand stigma in (re)emerging disease outbreak contexts, including the implications for affected populations and disease control. Our model aims to provide a comprehensive and structured understanding of this complex phenomenon. This is a critical step in building fit-for-purpose tools for current and future emergency responses.

## Methods

### Recruitment and data collection

We conducted once-off in-depth interviews of 30–60 minutes with 34 key stakeholders in the field of (re)emerging infectious disease outbreaks. Interviews were conducted between August 2023 and March 2024.

We applied maximum variation purposive sampling to include interviewees with experience in a wide range of settings, diseases, and specialist fields. We specifically sought stakeholders from each of the following groups: executive and senior leadership from local and international health emergency response organisations (including WHO, Médecins Sans Frontières, International Federation of Red Cross and Red Crescent Societies, and UNICEF), health policy leads from national ministries of health, risk communication and community engagement experts, frontline hospital staff, clinical and social science researchers, leads of affected community organisations and advocacy groups, and those with lived experience of the disease. We sought to ensure that no WHO regions or substantial outbreaks were missing from the sampling frame. To ensure the findings are current, we included respondents involved in recent outbreaks, such as the global mpox outbreak, Nipah virus outbreaks in Bangladesh, and the 2022 Ebola Disease outbreak in Uganda. Sample size was determined by thematic saturation.

We recruited participants through established contacts and international collaborators. We provided potential participants with information detailing the reasons for the research and nature of the study. All stakeholders contacted for an interview agreed to participate. We were unable to arrange interviews with two stakeholders due to availability constraints. All participants provided informed consent before starting the interview. Interviews were conducted one-on-one via Microsoft Teams using a pre-tested interview guide and securely recorded. All interviews were conducted by AP, a clinician and researcher who has training in qualitative methods and experience in clinical outbreak response independent of the contacted organisations and stakeholders. While the focus of the interviews was new and re-emerging outbreaks, we invited participants to also draw from their experiences with established or endemic infectious diseases, such as HIV, tuberculosis, and leprosy.

### Data preparation and analysis

Interviews were transcribed verbatim and reviewed by AP before being uploaded to NVivo Qualitative Data Analysis Software Version 14 and coded using an iteratively adapted codebook. The research team met weekly while conducting interviews and analysing data to discuss emergent themes.

**Fig. 1:**
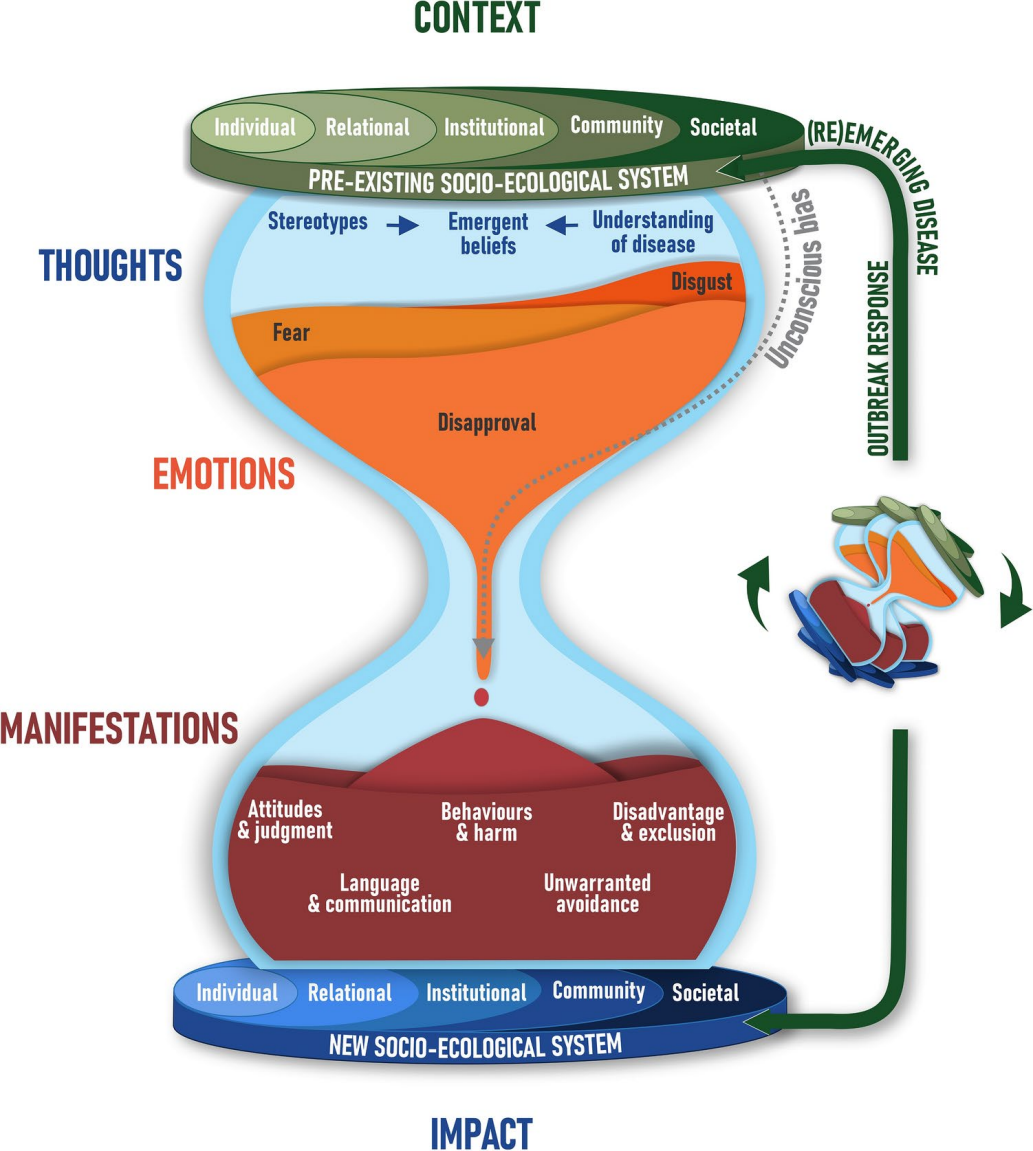
The hourglass stigma model. A conceptual model illustrating the stigma process through five domains.

We developed themes using mixed inductive and deductive thematic analysis. Once the major themes were developed from the data and agreed upon, the subthemes were created drawing on relevant social and behavioural theories. We reviewed a UNICEF synthesis of 25 social and behavioural theories^12^ and five widely recognised health stigma theories^7,11,13–15^ for applicability to the qualitative data. Theories were selected on the basis of explanatory power, simplicity, and best fit with the qualitative data. The theories applied to each domain are detailed in Table 1. Themes (model domains) and subthemes (factors) were then assembled into an explanatory analytic model, the hourglass stigma model (Fig. 1). Three participants reviewed the findings to ensure they were an accurate representation of the interview content.

**Table 1:** Social and behavioural and stigma theories applied to create subthemes within each domain of the hourglass model

Model domain/theme	Applied social and behavioural theories from literature	Core idea
Contextual factors	Socio-ecological model	Several concentric layers shape a person’s behaviours^16^
	Social norms theories	Behaviour may be determined by how others behave (empirical expectations), and/or how others expect the individual to behave (normative expectations)^17,18^
	Social network theory	Networks can influence individuals connected to others who engage in a particular behaviour, and who may persuade the individual to adopt a new behaviour^19,20^
	Media effects model	Agenda setting, priming, and framing by the media are powerful determinants of specific behaviours. This is commonly exploited for personal or group benefits in political, social, or economic battles^21^
	Jones stigma dimensions	Six features are commonly associated with stigma: concealability, course, disruptiveness, peril, origin, and aesthetics^13^
Cognitive processes	Health belief model	Self-efficacy, and cognitive biases can influence an individual’s perception of the seriousness of a problem, and the pros and cons of adopting a preventive behaviour^22^
	Decision-theoretic model of collective behaviour	Behaviours can either be non-social (when factual beliefs and personal normative beliefs are sufficient to drive an action) or social/collective, (when empirical or normative expectations are necessary to drive a behaviour^17^
	Attribution theory	The human mind is inclined to making attributions about what causes an event. These attributions are shaped by the perception of controllability and stability^23^
Emotional responses	Evolutionary theory of cognitive biases	“Errors” in human cognition may have emerged because they were helpful from an evolutionary viewpoint. For example, stereotyping may be advantageous for quickly forming opinions in certain circumstances^24^
Stigma manifestations	Reasoned action and planned behaviour model	Personal beliefs affect attitudes toward specific behaviours. These attitudes then influence intentions to carry out such behaviours^25^
	Jones and Corrigan stigma conceptual model	Stigma is triggered by stereotypes (negative beliefs) and manifests as prejudice (agreement with belief and/or negative emotional reaction) and discrimination (behavioural response)^14^
	Link and Phelan stigma theory	Stigma results from the co-occurrence of labeling, stereotyping, separation, status loss, and discrimination in the context of power^15^
Impact	Complex systems theory	There is complexity in the multicausality, multidimensionality and interdependency of cognitive, social and structural phenomena^26^
	Socio-ecological model	As detailed above^16^

Behavioural theories were selected from a UNICEF synthesis by Petit V^12^.

We identified key considerations that link factors within and across domains of the model and hold significance for outbreak preparedness. Examples of these key considerations are provided as quotes throughout the findings and a list of recommended questions relating to each is offered in the discussion.

#### Reporting standards

We have reported our methods and findings according to the Consolidated criteria for reporting qualitative research (COREQ) checklist (Supplementary Material Table 1)^27^.

## Results

### Characteristics of stakeholder participants

We conducted interviews with 34 purposively sampled stakeholders. The characteristics of the stakeholders are detailed in Table 2.

**Table 2:** Interview participant characteristics.

Participant characteristics	Participants No. (%), N = 34
Roles in outbreak response*	
Clinical care	20 (59)
Response coordination and operations leadership	18 (53)
Social science research	11 (32)
Patient advocacy	8 (24)
Psychosocial support	8 (24)
Policy making and governance	7 (21)
Clinical research	7 (21)
Training and capacity-building	6 (18)
Risk communication and community engagement	6 (18)
Health ethics	3 (9)
Dignified burial	2 (6)
Health economics	1 (3)
Disease-specific expertise/response experience*	
Coronavirus disease (COVID-19)	29 (85)
Ebola virus disease	21 (62)
HIV	15 (44)
Mpox	7 (21)
Marburg virus disease	6 (18)
Cholera	6 (18)
Influenza A (e.g., H1N1, H7N9, H5N1)	5 (15)
Nipah virus disease	5 (15)
Zika virus disease	5 (15)
Tuberculosis	5 (15)
Severe acute respiratory syndrome 1 (SARS-1)	3 (9)
Sexually transmitted infections	3 (9)
Dengue fever	3 (9)
Middle East respiratory syndrome coronavirus (MERS)	2 (6)
Lassa fever	2 (6)
Shigellosis	2 (6)
Measles	2 (6)
Plague	2 (6)
Meningococcal disease	2 (6)
Malaria	2 (6)
Pertussis	1 (3)
Leprosy	1 (3)
Anthrax	1 (3)
Hepatitis E	1 (3)
Non-polio enteroviruses	1 (3)
Regional experience*	
Africa	21 (62)
Europe	10 (29)
Americas	6 (18)
Western Pacific	6 (18)
South-East Asia	5 (15)
Eastern Mediterranean	2 (6)
Positionality in outbreak response	
Local responder (in-country)	13 (38)
International responder	9 (26)
Experience as both	12 (35)
Reported personal experience of relevant conditions*	
COVID-19	20 (59)
Influenzae A	3 (9)
HIV	3 (9)
Ebola disease	2 (6)
Mpox	1 (3)
Zika virus disease	1 (3)

*Participants may fit more than one category.

### The Hourglass model

The resultant cross-outbreak stigma model is conceptualised as an hourglass and detailed in Fig. 1. The model comprises five major domains, namely: context, thoughts, emotions, manifestations, and impact.

The hourglass structure is adopted to illustrate the broad array of contextual factors that contribute to stigmatisation processes which, in turn, can lead to a wide range of effects. The model embodies the notion repeated by multiple stakeholders that ‘outbreaks begin and end in communities’. The socio-ecological systems mirrored at the top and bottom of the model underscore that the impact of stigma from one outbreak shapes the environment for future outbreaks. That is, in a subsequent outbreak, the hourglass will turn, and the emergent socio-ecological system becomes the pre-existing context which drives or mediates stigma associated with the new outbreak.

We present our key findings organised by the domains of the model.

#### Domain 1: context

The model identifies three aspects of an outbreak context to be considered: the pre-existing socio-ecological system, the (re)emerging disease characteristics, and the outbreak response, all of which interact with one another. All contextual factors can be both drivers and mediators of stigma.

### Pre-existing socio-ecological system

**Fig. 2:**
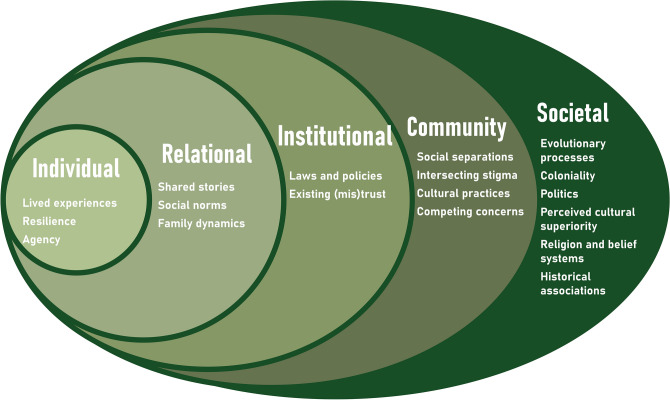
Factors within the pre-existing socio-ecological system. Levels are based on the socio-ecological model^16^.

The factors for consideration within the pre-existing system are detailed in Fig. 2 with illustrative quotes for each factor provided in Supplementary Material Table 2.

A key finding identified across stakeholder comments about pre-existing systems, was the impact of collective memory on (re)emerging stigma (Key Consideration 1). This includes collective memory of colonial interventions.

“Each country, each community, has their way of thinking and living. But there’s a history of eradication campaigns run by tropical medicine, you know, the monsoons of medical officers and massive colonial eradication campaigns. And I believe that there’s something about that stigmatisation. And it is something about the power imbalance in the dynamics. I think that’s still at stake nowadays in many countries.” – Interview 23, International response coordinator

“I remember there was a suspicion. The feeling was that the white people have brought Ebola here because they want something from us.” – Interview 14, Local Ebola psychosocial team lead

Collective memory was believed to similarly play out between recent disease outbreaks, with the stigma associated with a previous outbreak (e.g., SARS-1) impacting how people think about a new emergence, or even a new disease (e.g., COVID-19).

The stigma associated with not keeping to cultural practices and social norms (Key Consideration 2) was another common reflection on how outbreaks interact with pre-existing systems. Stakeholders ﻿who split their time across different contexts reflected on the differing norms around mask use in the early phases of COVID-19.

“It’s interesting because in Korea we have a kind of culture of wearing masks even before Covid. You can wear masks. People don’t care about that. But in the US they don’t have any culture like that. So they stigmatise people who wear masks.” – Interview 12, Local social scientist

All stakeholders with experience of Ebola outbreaks raised safe and dignified burials and the resultant restriction on cultural practices as an area of concern in relation to stigma.

“You’ve got a bunch of issues with coming into conflict with cultural practices. And I think that promotes a new type of stigmatisation. If washing of the body or touching the body are something that’s very much part of your culture, I think it’s very hard to give that up, even as a health message.” – Interview 20, International governance and ethics response actor

“In the beginning we were told strictly that there would not be any confirmation [of a death by relations]. The surveillance team would first go to that village and alert the people that a person has died and then we would organise and just go direct to the grave. And so that made people not feel comfortable because we were burying their people, but they have not confirmed the death.” – Interview 28, Local Ebola burial team member

### Emerging contextual factors

**Fig. 3:**
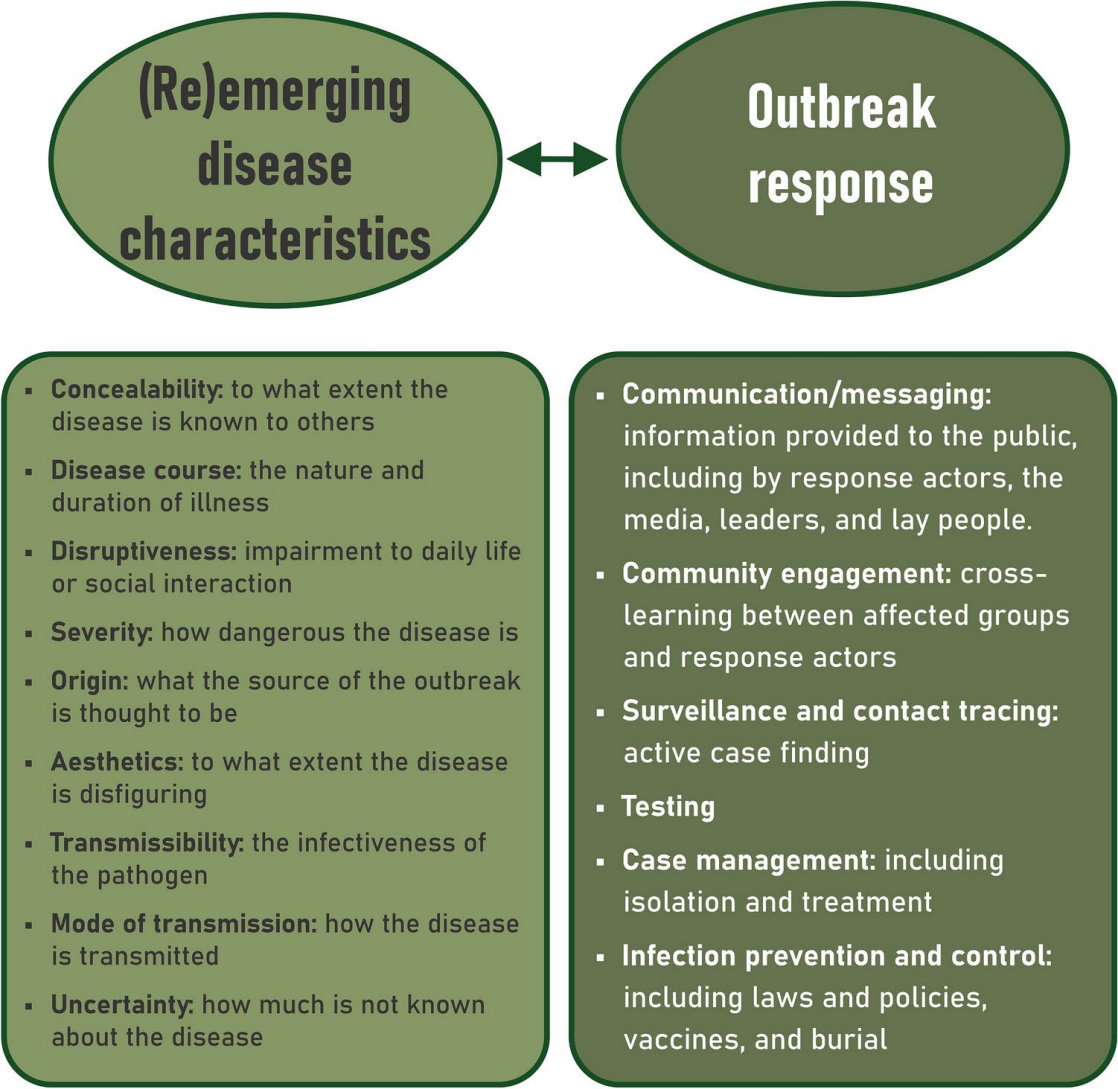
Disease and outbreak response factors that influence stigma. Categories are based on Jones’ Stigma Dimensions^13^ and the Outbreak Response Pillars^28^.

Disease characteristics associated with stigmatisation and outbreak responses believed to drive or mediate stigma are mapped in Fig. 3.

Participants described how disease characteristics and outbreak responses not only affect the pre-existing socio-ecological system (as illustrated in the burial practices example) but also impact one another. In particular, stakeholders commented on how outbreak responses can reduce disease concealability (Key Consideration 3). Various efforts to control an outbreak (including contact tracing, testing and notification, research involvement, and frequent healthcare visits) were noted to make those who had the disease more identifiable, facilitating stigmatisation.

“I think contact tracing fed into that sense that if you were known to be in contact with a person, that marked you in some or other way.” – Interview 1, Local testing centre coordinator

“If it’s a subtle disease that isn’t obvious to people, then taking part in a trial and someone seeing that you’re taking some medicines at home… you could identify yourself there.” – Interview 3, Local and international clinical responder/researcher

A few stakeholders commented on how test results (particularly of notifiable conditions) now mark people who may previously have had ‘invisible’ diseases.

“Some of the outbreaks that occur really bring back the stigmatising behaviours both at the individual and collective level related to infectious disease that don’t seem to have changed for thousands of years. Even if we now identify who is infected using a PCR test as opposed to a visual marker of disease. But I think it’s remarkable how persistent that can be.” – Interview 5, Local and international responder/ethicist

Many reflected on how the prospect of reduced concealability made communities more hesitant to embrace outbreak response measures.

“In my experience in Vietnam, in SARS-[CoV]-1 and bird flu, when we went to the communities to do contact tracing, the patients and their families were very reluctant to see public health officials because it was very stigmatising, and the families were often shunned by the community, and they weren’t involved in the communities. People weren’t talking to them and they lost jobs.” – Interview 9, International and local clinical responder/researcher

“I had people [with mpox symptoms] say to me “it’s not possible for me to be quarantined” or “it’s not possible for me to see someone, because if my parents knew about this and what I was being quarantined for… I'm not out to them. So I can’t go,” and in my head it makes sense that you can’t go, even though as a public health person, I'd say please go, it sounds like it’s a high chance that it could be mpox based on what you’re saying. But from a very human level and a community level, I think it’s ineffective for us to push things like that.” – Interview 32, Local public health practitioner and community advocate

Other stakeholders involved in mpox response similarly pointed out that these concerns about concealability were not limited to the disease itself, but also associated identities. This relates to another disease characteristic commonly raised in the context of pre-existing societal factors: mode of transmission.

Multiple stakeholders described how religious, political, and historical associations can result in moralisation of particular modes of disease transmission (Key Consideration 4). This phenomenon was noted for blood-borne diseases, those associated with drug-use, sexual transmission, and specific intermediate hosts.

“It was predominantly drug users who were involved with the anthrax outbreak in 2010. And of course, they’re just often treated as though, you know, they’re the lowest of the low.” – Interview 11, Local and international clinical responder and response coordinator

“From my experience the stigma comes to the play in a much bigger way when it’s a sexually transmitted condition. So when it involves sex, it automatically comes with stigma around certain communities such as gay, bisexual, [men who have sex with men] communities. All because sex is still taboo itself.” – Interview 7, Local HIV and mpox advocate with lived experience

“With Nipah when I was a child, it was very much considered a pig disease. And there were lots of tensions at the time between people who ate pork and didn’t because of religious reasons in Southeast Asia. This was also the time of major race riots. Quite a few people like me were being massacred in Indonesia around that time and tensions were therefore high. So I think the association of Nipah virus with pigs as an intermediate host was quite politically charged.” – Interview 10, Local and international clinical responder/researcher

#### Domain 2: thoughts

**Fig. 4:**
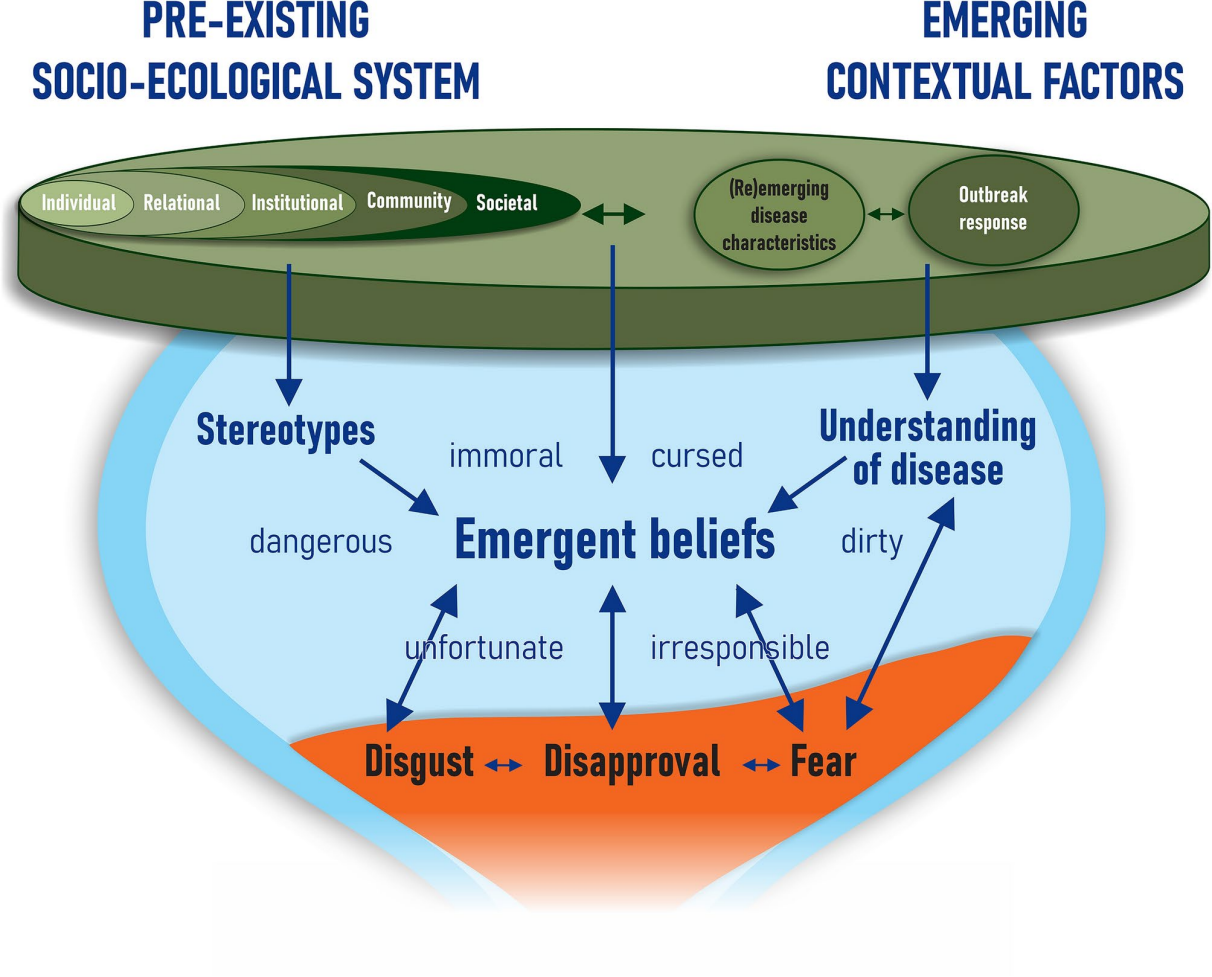
Cognitive aspects of the stigmatisation process in relation to context and emotions.

As illustrated in the hourglass stigma model, the contextual factors combine to result in various thoughts about the disease and those affected. Multiple interviewees described how stereotypes (from the pre-existing context) and understanding of the disease (from the emerging context) inform these thoughts. These cognitive pathways, as well as the common beliefs (e.g. affected people are immoral, cursed, dirty) identified in the qualitative interviews, are summarised in Fig. 4.

The stakeholders’ shared concern that those who cannot adhere to prevention measures may unjustly be marked as irresponsible (Key Consideration 5) is an example of how pre-existing context (social divides) and outbreak response combine to form an emergent belief.

“You become ostracized or stigmatised because you cannot follow the policy. But the policy is not appropriate for you in your setting, right?” – Interview 20, International governance and ethics response actor

“Zika was so hard because the communication was so poor and it was very much, well just don’t get pregnant. Like that’s even possible. And so stigma from not ‘following instructions’ from the Ministry of Health [meant that getting Zika] was “are you going to be a bad mother? Are you being selfish? Are you just ignorant?” The stigma was you’re a fool or you’re dirty, which… this idea of hygiene and dirt just goes across any level of stigma” – Interview 4, Local and international community engagement and public health specialist

As suggested by the last quote, a prominent concern was that hygiene-dominant public health messages can result in beliefs that people who get the disease are ‘dirty’, exacerbating social divides (Key Consideration 6). This was particularly noted for specific disease types such as cholera.

“In something like Ebola or Covid, there are, of course, social and economic factors, but everyone more or less can get these things. Whereas for cholera, if you’re rich and have toilets and water, it’s just not going to happen, or at least very unlikely. And so, you see much more of that piggybacking of saying, well, these people are dirty and therefore they get cholera. And people who have cholera are dirty people, and around and around you go.”  – Interview 16, International community engagement and outbreak response coordinator

The cognitive aspect of stigma most commented on by stakeholders was the relationship between knowledge and stigmatising beliefs. The examples given showed that there is not a linear relationship between knowledge of a disease and amount of stigma (Key Consideration 7).

Most stakeholders suggested that this was because the presence of multiple contextual mediators meant that disease knowledge is not the only factor affecting emergent beliefs.

“We still just assume that people are logical and rational creatures and that therefore means that they will make their decisions based on the best health information. And people can be hyper rational, but religious information, social information, cultural information, power dynamics, all of these things that are playing into their decision making … And I think stigma comes into that in that we just assume, well, if I tell you more information about this disease, you will make the right choice.”  – Interview 16, International community engagement and outbreak response coordinator

The presence of ongoing questioning despite information was offered as evidence of the limitations of knowledge when it comes to stigma reduction.

“If you live within a certain cultural model, you have two questions you always ask, two ‘Whys’. The ‘Why empirical’ is what we all use. I'm crossing the road, a car knocks me down, I break a leg. You know if the car didn’t hit me, I wouldn’t break my leg. That’s clear enough for anybody to understand, right? And people do believe that. But then there’s that back thinking, oh, why me? Why today? Why? Right? That’s the ‘Why two’… And I’m not saying that we should turn medical doctors into spiritualists, but at least we need to take that seriously. And not ignore it completely.” – Interview 21, Local and international health ethicist

Some interviewees drew the connection between the above consideration, and another: that people may use a disease as a reason to further stigmatise high risk groups they morally disagree with (moral-piggybacking) (Key Consideration 8). An interviewee gave an example from the 2022 mpox outbreak:

“The comments I saw on Twitter were in the context of mpox, but they’re the kind of comments that you get that are actually driven by underlying homophobia rather than anything else. But of course, that is quite important when you’ve got one disease that is affecting a particular group and those people happen to be gay.” – Interview 3, Local and international clinical responder/researcher

#### Domain 3: emotions

The emotional domain of the hourglass model reflects the idea that emotional responses can be derived from cognitive processes (which can change and be mediated). However, it also highlights that emotional responses (such as fear) can be triggered by automatic negative reactions and therefore difficult to eliminate completely (Key Consideration 9).

“We get anxious and we get afraid because it’s our way of making sure we stay out of danger. So it’s hard to work out what the solution to that is.” – Interview 8, Local mpoxnon-governmental response coordinator and advocate

“We are humans and once we identify anything that could threaten us, of course we try to distance ourselves from it.” – Interview 27, Local Ebola clinical responder and survivor programme developer

The similarities in these comments suggest that this key consideration holds true across vastly differing outbreak contexts.

Another prominent focus within the interviews was the concern that appealing to fear for the purpose of enhancing adherence to preventive measures may have undesired consequences (Key Consideration 10). From their varying perspectives, stakeholders explained how intentional fear appeals in outbreak messaging may result in reduced rationality and compassion:

“I think the truth can be scary and we have to tell people the truth. But intentionally scaring people, I don’t think it’s ethical, and I also just don’t think it gives results. If you’re being purely machiavellian, I don’t think it works because scared people do not make good decisions. People run the wrong way in fires. People make stupid decisions in disasters, fear does not make us more compassionate people and it does not make us more effective decision makers. We also know that fear wears off over time… so if [an outbreak response] is based on people being afraid of this threat, if the threat lasts longer than the adrenaline surge does, then we just become accustomed and we move on, even though the risk has not actually decreased.”  – Interview 16, International community engagement and outbreak response coordinator

Other stakeholders described how this fear can drive false disease narratives and conspiracy theories:

“To be rationally fearful is one thing. But most of our fears are not rational. This gives a good breeding ground for conspiracy theories, and myths, because it breaks down the trust system. And once people are under a form of repression they are more likely to buy into stories.” – Interview 21, Local and international health ethicist

#### Domain 4: Manifestations

**Fig. 5:**
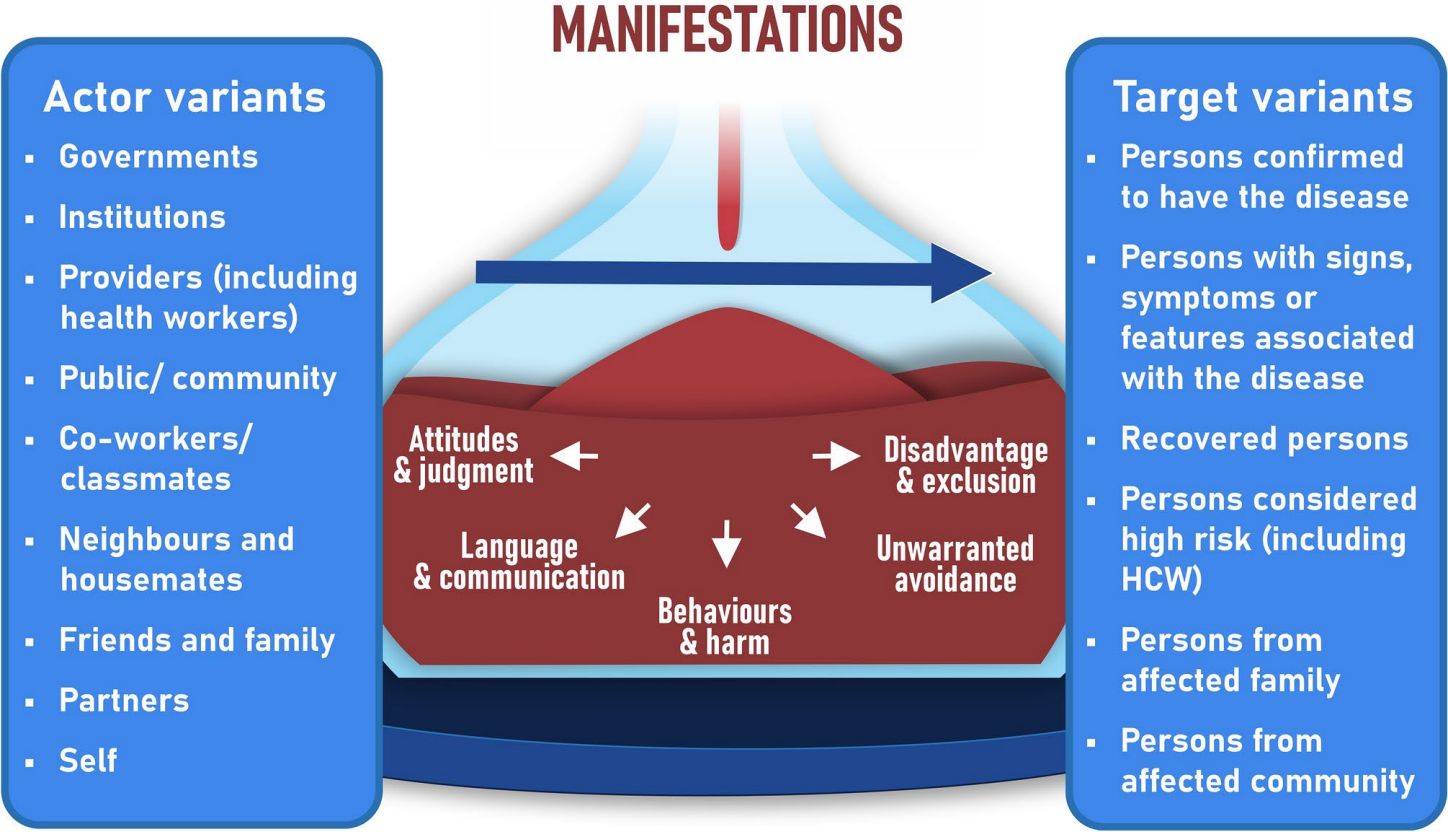
Stigma manifestations with actor and target variants. Actors refer to those enacting stigma, while targets are the individuals or groups affected.

When asked about how stigma was visible in their settings, stakeholder responses demonstrated that stigmatisation in outbreak contexts extends beyond discrimination against infected individuals (Key Consideration 11). We charted the range of stigma manifestations in Fig. 5 with examples provided in Table 3.

**Table 3:** Examples of different stigma manifestations. HCW = healthcare worker.

Quotes for each action-based manifestation	Actor	Target	Disease
Attitudes and judgement			
“It’s often households, I felt, that you’re culpable as a family. Particularly so in bird flu, H5N1, anyway, a lot of children are affected and their families felt the impact of stigma” – Interview 9	Government, community	Family, household	Avian influenza
Language and communication			
“I met some survivors from way back in Gulu outbreak. That was 2001. And one of them was telling us that he stood for some political positions and they were saying his opponents were referring to him as Ebola. This is a candidate for some position, but you’re not even using their name. They’re just saying, oh, that Ebola candidate, that Ebola candidate.” – Interview 13	Community	Recovered persons	Ebola disease
Behaviours and harm			
“Again, there was geographic association… that it had come from China. So there were a lot of targeted racist attacks in London. So there was a young student from Singapore who was beat up for it. I had patients who refused to see me in the hospital. I had senior staff members say to me that it was probably better I dealt with the Covid patients because, you know, subtext, people think that you might have caused it sort of thing. So that was… that was quite tough.” – Interview 10	Public, co-workers	Persons with associated traits	COVID-19
“My first work with cholera outbreaks, um, I remember cars being stoned. No access to certain villages because we were ‘bringing the disease in’… And we always feared when going to a cholera outbreak that without good sensitization to the community, we were going to pay the price.” – Interview 24	Community	International response workers	Cholera
“One of our colleagues was investigating the virus, investigating Nipah. And they actually locked her in a house in a village. And then we needed to go with police and other local administrator to get her back. And they locked her there because they thought she was spreading rumors about this new virus, this new thing that they’d never heard about and they were really scared.” – Interview 31	Community	Local response workers	Nipah virus disease
Unwarranted avoidance			
“I think I just got ashamed when I was out of the Ebola Treatment Unit and I was home. I spent two weeks at home. I would never come out of my house because I was fearing being out for the public to see me. I was ashamed of Ebola. I felt like, how will people take it?” – Interview 30	Self, community	Recovered persons	Ebola disease
“I remember a story from a man in Indonesia who was saying he had been treated and he was no longer contagious, but nobody would be willing to pray next to him at the mosque. So he rather decided that he didn’t want to go anymore. And so you get this kind of internalized stigma kicking in, which basically means that the net effect on people’s social functioning can be quite similar as when they are ostracised.” – Interview 6	Community	Recovered persons	Leprosy
Disadvantage and exclusion			
“But for the survivors, of course, it was a cost because landlords were chasing people from their houses.” – Interview 25	Providers (landlords)	Recovered persons	Ebola disease
“When we were asking for money to support people there was this rhetoric of, “Oh, we need to be careful what we do with taxpayers money”. And the point that was made time and time again was that gay, bisexual men are paying tax as well. They are. They’re not this separate entity outside of the rest of the population. They’re also funding the National Health Service. So when they need it, they should be able to benefit from it.” – Interview 8	Government, institutions	Affected communities	mpox
“And of course there are some things, like you’re exporting goods, the agriculture products that could be coming from Rwanda and some people would shy away from them. Well, this is coming from a district that has Ebola, the district that is on a lockdown. So I don’t think I want to take this.” – Interview 13	Public, consumers	Associated communities	Ebola

Participant quotes illustrate the different manifestations of stigma across a range of infectious disease outbreaks. The ‘Actor’ column indicates who enacted the stigma, while the ‘Target’ column specifies the group affected.

These manifestations of stigma are thought to largely arise from the cognitive and emotional pathways described, however stigma may be enacted directly from contextual factors, without negative beliefs or emotions, due to unconscious bias (Key Consideration 12). This is illustrated by the dotted line in Fig. 1. Stakeholders referred to the use of terms such as ‘Chinese virus’ in COVID-19 as an example of how people may unknowingly propagate stigma, leading to unintended adverse effects.

“We are oftentimes not really aware of how stigmatising our language can be.” – Interview 17, Local social scientist

#### Domain 5: impact

**Fig. 6:**
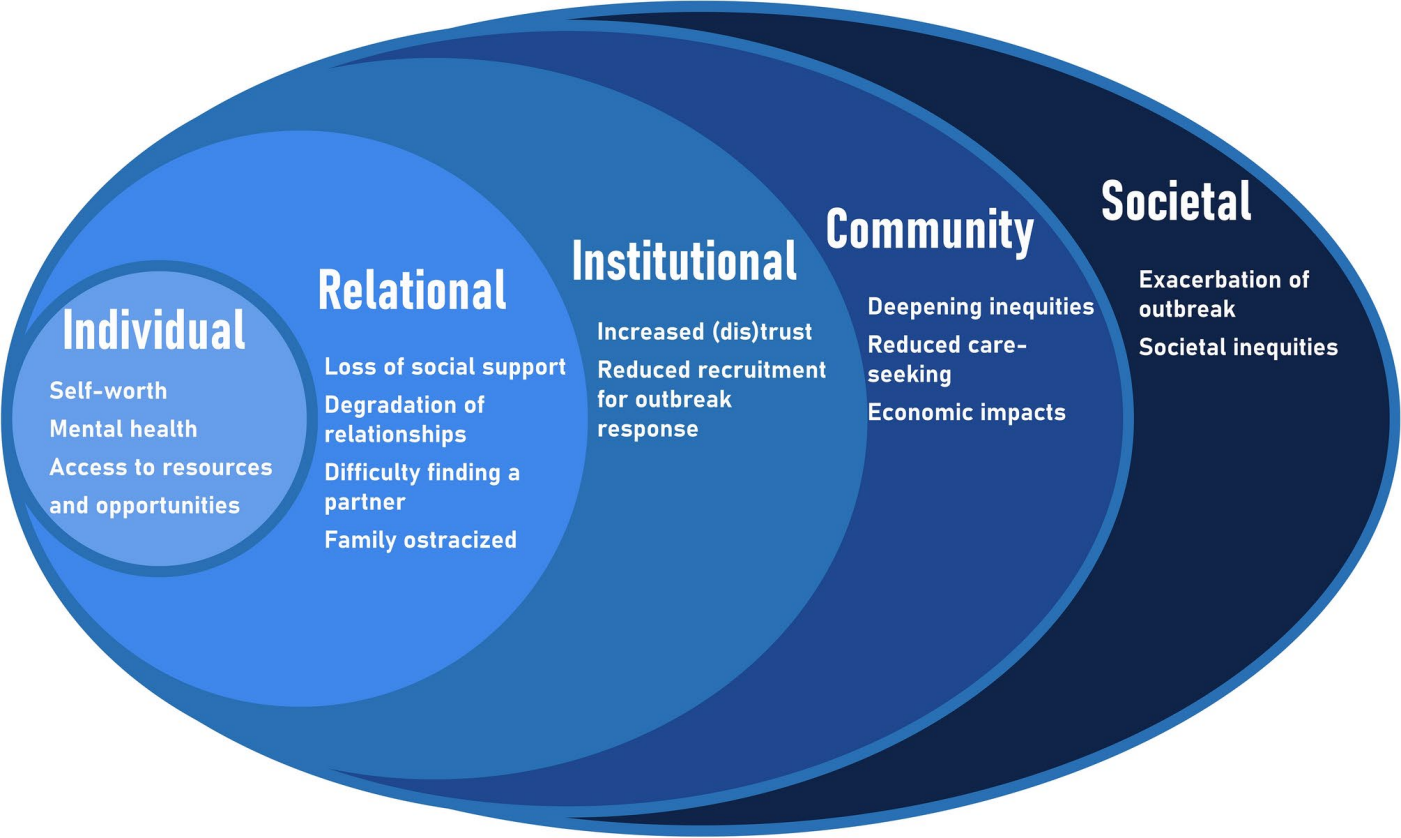
Impacts of stigma resulting in the emerging socio-ecological system. Levels are based on the socio-ecological model^16^.

The final model domain is concerned with the impact of stigma. Impacts described by stakeholders could be mapped to all socio-ecological levels (Fig. 6). This creates a new socio-ecological system which changes the profile of stigmatisation in future outbreaks.

On an individual and relational level, it was evident from the interviews that stigma can have psychosocial consequences that outlast the disease (Key Consideration 13).

“In our settings, in Africa, and Uganda in particular, we’ve not really embraced mental health. And it is something that is really important and we need to try to embrace it because a person who has recovered from Ebola himself or herself has to be mentally prepared, mentally know that ‘I am okay. I have recovered. I'm totally fine. I can go back to normal function.’ We’ve seen a lot of survivors who have been discharged and then turn [to drugs].”  – Interview 13, Medical doctor and Ebola survivor

Multiple stakeholders emphasised that these psychosocial consequences of stigma reduce access to treatment and should therefore be considered a social determinant of health.

At an institutional level, comments returned to Key Consideration 1 about collective memory, noting that stigma manifestations can have a lasting impact on trust in institutions (Key Consideration 14). It was suggested that the solution to this issue is not finding ways to automatically increase community trust but first improving the trustworthiness of response actors.

“I worry a lot when people want to increase community trust without increasing their own trustworthiness.” – Interview 5, Local and international responder/ethicist

Another stakeholder termed community members’ scepticism ‘healthy mistrust’. It was noted that the level of (mis)trust often varies over the course of an outbreak.

“Initially [concerns about stigma] made people not want to give away their loved ones, not to report them to the health care workers. They would prefer to run with them to the traditional health worker. They’d prefer to hide them in the houses and treat them from their self-medication. But, again, this came with negative effects to the same families… And eventually they started opening up to the health care workers and this became more acceptable within the community. And actually, we began to see big numbers of survivors coming back to their communities. Then people started looking at the isolation facilities as the only areas where they could survive, something good.” – Interview 15, Local clinical outbreak responder

This example shows how trust in institutions can improve over time facilitated by effective treatments and proven trustworthiness.

Stakeholders provided examples of how stigma may worsen outbreak control (Key Consideration 15). As suggested throughout the key findings, stigma can reduce timely care-seeking, affecting the ability to rapidly contain outbreaks.

“I had two friends. I was with them and the patient [who had Ebola]. One was the first person to show up the signs. But he never turned up to go to the hospital. We buried this friend of ours who had died of Ebola on Tuesday, he turned up on Wednesday at my facility and was expressing the signs. I, myself, I told him to turn up and go to the hospital, to the regional referral. He refused. He said “I cannot go there. How will the community hear that I’m having that disease?”” – Interview 30, Local nurse and Ebola survivor.

“And that was one of the reasons why people were not coming to test, even though they had symptoms, they would not tell anyone, because if they had symptoms [of COVID-19] and they get tested, the police would lock the house. So many of those who had symptoms, they’d actually go to the market, go to the common place. And that’s why. They were hiding from testing so people couldn’t tell that they got this disease.” – Interview 31, Local clinical researcher/outbreak responder.

Stigma was believed to make recruitment of response personnel more difficult.

“For example, when I said to my family, I want to be an infectious disease doctor, I think their first reaction was, “but that’s dirty work”” – Interview 10, Local and international clinical responder/researcher

“Healthcare workers that were involved across the different response pillars, IPCs in my case, there was a reluctance on the part of the community to engage with them, to freely socialise with them, and that in a way, I think disillusioned people from being part of the responders. I have some cases where people felt, well, it’s not worth it to really be a responder here because my family is very uncomfortable.” – Interview 14, Local Ebola infection prevention and control (IPC) trainer

Those involved in safe and dignified burials said their teams were similarly limited by the lack of personnel willing to do this work.

## Discussion

The practical implications of stigma in (re)emerging infectious disease outbreaks are profound. By integrating established behavioural science theory and stakeholder insights, we built this novel conceptual model to be a bridge between theory and practice in the field.

A consequence of the current gap between outbreak stigma-related interventions and underlying theory is the indiscriminate reliance on default interventions, such as addressing any form of stigmatisation with an education campaign, irrespective of what the root of the behaviour may be^12^. This raises concerns about the judicious allocation of scarce resources and the effectiveness of programmes. It also ignores the fact that as outbreak responders, we may be partially responsible for some of the stigmatisation, and it may be more effective to adapt outbreak interventions rather than independently trying to address the resultant stigma.

This study illustrates how pervasive stigma is across a range of (re)emerging outbreaks. It highlights the need to consider stigmatisation of groups who may be associated with the virus without being infected themselves (sometimes termed courtesy or associative stigma). This stigma model compliments and builds on the broader Health Stigma and Discrimination Framework (HSDF)^11^ but differs in its conceptualisation in a few key aspects: firstly, the hourglass framework is unique in that it offers an exploration of how factors interact with one another. Secondly, while the HSDF considers contextual factors necessarily drivers or facilitators of stigma, the hourglass framework suggests that contextual factors may also be protective against stigmatisation. For example, a context of community norms that are accepting of difference can minimise stigma in an outbreak. Finally, the hourglass model places institutions within communities in the socio-ecological system, rather than external to them, highlighting that these institutions (while often seen as larger than a community) fundamentally operate within complex community structures alongside other actors.

A major challenge in outbreak response is the ability to systematically assess and address stigma in real-time. Stigma-related behaviours are often dismissed as social byproducts rather than core considerations in public health decision-making. However, stigma can fundamentally shape outbreak trajectories, influencing health-seeking behaviours, public trust, and adoption of preventative measures. Without structured approaches to integrating stigma considerations into response strategies, interventions risk being ineffective or even counterproductive.

Our intention in developing the hourglass model was for key stigma considerations to be readily applicable in practice, facilitating nuanced reflection on stigma and the ways in which outbreak response efforts can either contribute to or prevent it. These reflections currently typically occur retrospectively or late in the course of an outbreak. Table 4 offers an example of how the model can be used in practice with a list of question prompts to assist with structured consideration of stigma during (re)emerging outbreaks.

**Table 4:** Stigma domains, key considerations, and question prompts for outbreak responders.

Domain	Key considerations	Question prompts for outbreak responders
Context	(1) The impact of collective memory on (re)emerging stigma (2) The stigma associated with not keeping to cultural practices and social norms (3) Outbreak responses can reduce disease concealability (4) Religious, political, and historical associations can result in moralisation of particular modes of disease transmission	· Which previous outbreaks or events may affect how the community perceive this outbreak? · What cultural practices and social norms may be disrupted by outbreak containment efforts? Is there a way to minimise this disruption? · Can outbreak responses be adjusted to improve confidentiality while still reducing the spread of the disease? · Have the social practices associated with the mode of transmission previously had moral judgement associated with them?
Thoughts	(5) Those who cannot adhere to prevention measures may unjustly be marked as irresponsible (6) Hygiene-dominant public health messages can result in beliefs that people who get the disease are ‘dirty’, exacerbating social divides (7) There is not a linear relationship between knowledge of a disease and amount of stigma (8) People may use a disease as a reason to further stigmatise high risk groups they morally disagree with (moral-piggybacking)	· Are policies appropriate for all socio-economic levels of society? · What contextual factors may be contributing to emergent beliefs? Is community listening occurring alongside education? · What information sources are trusted by the community? · Are disease prevention messages appropriate for all socio-economic levels of society? · Are any communities/groups likely to experience compound stigma due to the risk communication messages? Can the messaging be redesigned to reduce this stigma? What can be done to support these groups?
Emotions	(9) Emotional responses (such as fear) can be triggered by automatic negative reactions and therefore difficult to eliminate completely (10) Appealing to fear for the purpose of enhancing adherence to preventive measures may have undesired consequences	· Is the fear associated with the disease proportionate to risk? · Is public health messaging purposefully appealing to fear? Could this impair rationality or foster misinformation? · What additional risk-mitigation actionable steps can be recommended alongside messages that may induce fear? · Does the disease trigger a ‘disgust’ response? How can this be reframed in messaging?
Manifestations	(11) Stigmatisation in outbreak contexts extends beyond discrimination against infected individuals (12) Stigma may be enacted directly from contextual factors, without negative beliefs or emotions, due to unconscious bias and social norms	· Which groups may be stigmatised in association with the outbreak? Are families, healthcare workers, and associated communities at risk of stigmatisation too? · How does stigma typically manifest in the affected community? · What measures can be introduced to minimise exclusion of recovered persons? · How might unconscious bias, including language choice, be contributing to stigma?
Impact	(13) Self-stigma may have psychosocial consequences that outlast the disease (14) Stigma manifestations can have a lasting impact on trust in institutions (15) Stigma may worsen outbreak control	· Are psychosocial support mechanisms available? Do they directly account for and aim to address stigma for all affected groups in addition to survivors (e.g., family members and response workers)? · How could the trustworthiness of institutions be enhanced, demonstrated, and maintained? · Is stigma likely to be reducing healthcare seeking behaviour? Who are the alternative informal carers in the setting and how can they be better equipped?

This table illustrates how the hourglass model can be applied in practice, offering structured question prompts to help outbreak responders assess and address stigma during outbreaks.

By explicitly addressing stigma through structured reflection, response actors can anticipate potential challenges and adapt their interventions accordingly. Ensuring that stigma is recognised as a core element of outbreak response may contribute to more equitable, effective, and community-centred strategies.

### Limitations

This study design was limited by the requirement for interviewees to have access to the internet. There was an over-representation of stakeholders who have experience of certain diseases (e.g., COVID-19, Ebola) and regions (e.g., African region) due to the frequency and extent of relevant outbreaks and the contacts available to the research team.

The model also has limitations. We acknowledge that this hourglass model will not capture all the intricacies of a social phenomenon as complex as stigma. It is also possible that certain factors included in the model (e.g., emotions such as disgust) may not be relevant across all outbreaks.

### Future work

The conceptual model can be used to design and implement targeted stigma reduction interventions by a range of outbreak responders and organisations. This model is currently being used as the theoretical underpinnings for the development of a cross-outbreak stigma assessment tool. It would be useful for the model to be tested across regions and diseases, particularly in the early phases of a (re)emerging outbreak.

## Conclusion

Our model provides a systematic approach for comprehending and addressing stigma in emerging infectious disease outbreaks. It can be used for local and international outbreak response planning, health policy development, and research.

## Supplementary Information

Supplementary Information.

## Data Availability

The raw interviews from this study are not available due to risk of re-identification. The model vectors are available from the corresponding author on reasonable request.

## Abbreviations

COVID-19: Coronavirus disease 2019
HSDF: Health stigma and discrimination framework
IPC: Infection prevention and control
MERS: Middle East respiratory syndrome coronavirus
SARS-CoV-1: Severe acute respiratory syndrome coronavirus 1
WHO: World Health Organization
