# Supplementary material for: An hourglass model for conceptualising stigma in infectious disease outbreaks
Source: Sci Rep. 2025 May 2;15:15339. doi: 10.1038/s41598-025-98995-w (PMC12048598; doi:10.1038/s41598-025-98995-w)
Supplement: Supplementary file 1 — Supplementary Information. [file 41598_2025_98995_MOESM1_ESM.pdf]

An Hourglass Model for Conceptualising Stigma in Infectious Disease Outbreaks

Supplementary Material

Supplementary File 1: COREQ checklist

Consolidated criteria for reporting qualitative studies (COREQ): 32-item checklist

Developed from:

Tong A, Sainsbury P, Craig J. Consolidated criteria for reporting qualitative research (COREQ): a 32-item checklist for interviews and focus groups. International Journal for Quality in Health Care. 2007. Volume 19, Number 6: pp. 349 – 357

Item No	Guide Questions/Description	Reported on Page #
**Domain 1: Research team and reflexivity**		
**Personal Characteristics**		
1. Interviewer/ facilitator	Which author/s conducted the interview or focus group?	Pg 4
2. Credentials	What were the researcher's credentials? E.g., PhD, MD	Pg 4
3. Occupation	What was their occupation at the time of the study?	Pg 4
4. Gender	Was the researcher male or female?	NA
5. Experience and training	What experience or training did the researcher have?	Pg 4
**Relationship with participants**		
6. Relationship established	Was a relationship established prior to study commencement?	Pg 4
7. Participant knowledge of the interviewer	What did the participants know about the researcher? e.g. personal goals, reasons for doing the research?	Pg 4
8. Interviewer characteristics	What characteristics were reported about the interviewer/facilitator? e.g. Bias, assumptions, reasons and interests in the research topic	Pg 4
**Domain 2: study design**		
**Theoretical framework**		
9. Methodological orientation and Theory	What methodological orientation was stated to underpin the study? e.g. grounded theory, discourse analysis, ethnography, phenomenology, content analysis	Pg 5
**Participant selection**		
10. Sampling	How were participants selected? e.g., purposive, convenience, consecutive, snowball	Pg 4
11. Method of approach	How were participants approached? e.g., face-to-face, telephone, mail, email	Pg 4
12. Sample size	How many participants were in the study?	Pg 4
13. Non-participation Setting	How many people refused to participate or dropped out? Reasons?	Pg 4

Item No	Guide Questions/Description	Reported on Page #
14. Setting of data collection	Where was the data collected? e.g., home, clinic, workplace	Pg 4
15. Presence of nonparticipants	Was anyone else present besides the participants and researchers?	Pg 4
16. Description of sample	What are the important characteristics of the sample? e.g. demographic data, date	Pg 7-8
**Data collection**		
17. Interview guide	Were questions, prompts, and guides provided by the authors? Was it pilot tested?	Pg 5
18. Repeat interviews	Were repeat interviews carried out? If yes, how many?	Pg 4
19. Audio/visual recording	Did the research use audio or visual recording to collect the data?	Pg 4
20. Field notes	Were field notes made during and/or after the interview or focus group?	Pg 4
21. Duration	What was the duration of the interviews or focus group?	Pg 4
22. Data saturation	Was data saturation discussed?	Pg 4
23. Transcripts returned	Were transcripts returned to participants for comment and/or correction?	Pg 5
**Domain 3: analysis and findings**		
**Data analysis**		
24. Number of data coders	How many data coders coded the data?	Pg 5
25. Description of the coding tree	Did the authors provide a description of the coding tree?	Pg 5
26. Derivation of themes	Were themes identified in advance or derived from the data?	Pg 5
27. Software	What software, if applicable, was used to manage the data?	Pg 5
28. Participant checking	Did participants provide feedback on the findings?	Pg 5
**Reporting**		
29. Quotations presented	Were participant quotations presented to illustrate the themes/findings? Was each quotation identified? e.g., participant number	Pg 20-21
30. Data and findings consistent	Was there consistency between the data presented and the findings?	Pg 20-21
31. Clarity of major themes	Were major themes clearly presented in the findings?	Pg 20-21
32. Clarity of minor themes	Is there a description of diverse cases or a discussion of minor themes?	Pg 20-21

Supplementary Table 2: Illustrative quotes for pre-existing contextual factors

**Contextual factors**	**Illustrative quotes**
**Societal**	
Evolutionary processes	*We are humans and once we identify anything that could threaten us, of course we try to distance ourselves from it.* – Interview 27
Coloniality	*I remember there was a suspicion. The feeling was that the white people have brought Ebola here because they want something from us.* – Interview 14
Politics	*This message was echoed by some of the opinion leaders, like members of parliament who are saying, no, this is a hoax, this is a hoax for the government to get money from donors.* – Interview 14<br><br>*Whether these things are going to be labeled and perceived as a pandemic are also very politically and socially constructed.* – Interview 31
Perceived cultural superiority	*There was also the "we can't use masks" at the beginning of the pandemic because "culturally we're different". It was kind of this cultural superiority, this idea that people were somehow different, even though I think Covid, as it swept across the world, demonstrated that we're not very different at all.* – Interview 10
Religion and belief systems	*For instance, where there were clusters of infections... there was an allegation that the Muslim community exhumed a body that had been buried by a burial team to perform the religious rituals of burial. So that was the point at which we brought in the religious leaders to fully understand the risk of not following the due process. I think this is important: had the Muslim leaders been adequately informed and prepared and convinced that actually the right religious burial rites had been followed or there had been efforts to minimize contamination of the community to a level that's acceptable, we probably would not have seen those clusters of infection where we've had as many as 20 new cases in one day. So I don't think we adequately engaged them.* – Interview 14

Historical associations	*So, for HIV, I think there's a very strong historical, cultural, political aspect to it. And, I guess, I refer to, in the 1980s, what the different forms of conflated narratives were, around drug use, certain kinds of sexual identities, you know, even then, and we still hear these narratives today, so I think in that sense, those social determinants have political and historical roots to them.* – Interview 31<br><br>*So I think blood does have a broader meaning than just as a bodily fluid. It holds all sorts of ritualistic and cultural ideas about cleanliness and lineage. And, you know, it's a very, very complex substance and contamination by blood and we think about that everywhere in the West, you know, it has been historically stigmatised.* – Interview 2
**Community**	
Social separations	*I think in the South African context, it's always complex. But there was a very strong sense of, 'poor people live in crowded communities, poor people have a particular demographic in our country, and they were the ones that were driving the pandemic' from people who were able to protect themselves behind high walls and in smart houses, even though they were also getting sick.* – Interview 1<br><br>*So in Congo you have basically two primary communities. You have the Indigenous community and the non-Indigenous community, and survival rates were much lower for the Indigenous community because they're slower to seek care and that is a result of stigma, but not stigma about Ebola, about being a person who experiences stigma within that community and discrimination. So yeah, it blurs into the social determinants of health at some point.* – Interview 16
Intersecting stigma	*There was definitely an obesity-related stigma I think, that you know people have done it to themselves and made themselves sick and put other people at risk because they hadn't been able to control their weight.* – Interview 1

	This virus will show up all the weaknesses in your systems. Every system, every weakness. At some point it's about to stare you in the face because that's what outbreaks do. And that's exactly what SARS-CoV-2 did. Of course it did. And part of that was the vulnerable populations. – Interview 11
Cultural practices	*This I believe, also: that the western gaze onto the people who are affected by these diseases is limited. We do not realize what the real reason for stigma is… because I don't think that people feel fear or stigmatised because of the disease in itself, always. I'm not denying that is there, but in fact, sometimes what they fear most is stigma by their own community because they do not play the role for which they are meant to under their social codes, which is to take care of your sick relatives until death, you know, and even beyond that. What I mean is that what is stigma for us is not necessarily the stigma they really feel. You know, so for them, they are embarrassed, not because they are Ebola infected, but because in front of their community, because they couldn't take care of their people.* – Interview 23
Competing concerns	*In Sierra Leone, you have one of the highest maternal mortality and infant mortality rates in the world outside of Ebola. A mother has five, six kids. They live upcountry. They do not have access to healthcare on a simple basis. They have to survive the way they can through caring for their relatives within a small community, and all of a sudden they are told that you may not care for your relatives because you're going to become infected. So we arrived, good intentioned people for sure. But you know that mother, I mean, she's already lost several infants. Life or death depends on the community. Her community, not whatever outside massive international outbreak response might appear all of a sudden in her environment. So it's like, look, I'm going to die one way or another and in this country, in this life, me and my people are at very high risk of dying from any other disease, any other disease, because there's no prevention, there's no*

	access to health care. So why should I get that excited, you know? – Interview 23
**Institutional**	
Laws and policies	*The border closures to Africa in response to Omicron were, I think, an example of where prejudicial views about African populations and views about Covid coincided.* – Interview 5<br><br>*So in the UK when we are talking about when Section 28 happened, when you weren't allowed to talk about your gender and sexual orientation in schools. So that automatically created this value of, you know, oh, being gay, being LGBTQ+ is a bad thing. So when a condition impacts those communities, automatically people think that, oh, of course, gay communities. So this is very much related.* – Interview 7
Existing (mis)trust	*I think there's something about trust in science and whether people have a background where they feel that they've been treated fairly.* – Interview 18
**Relational**	
Collective memory	*In Southeast Asia, where these events have happened in the past, people are usually more prepared in a way or sometimes more cautious. And in the airports, for example, it's almost customary to wear a mask. So in the beginning, people that were early adopters in a way, and wearing masks, they were also being looked at in a way that was not as much... public stigma.* – Interview 17
Social norms	*The reliance on social groups or the social coherence and is it a rather individualistic culture or not? Things like that play a role in how you conceptualize the idea of stigma or the expectations of your social surroundings. And this might also be taken into account when thinking about stigma for infectious diseases from a global perspective.* – Interview 17<br><br>*You behave in a way because you think your community behave in that way.* – Interview 2

Family dynamics	*Initially the African way of doing things is if you lose your parents then somebody like an uncle actually takes you in to live as his child. And that's what was happening in West Africa. People were dying and then the children were moving to other families and from there they would start infections and so on and so forth. So what developed out of that now, nobody wanted to look after anybody's child even if they were discharged from the ETU. Nobody wanted to take care of them. But with time now, they have got to learn that actually when somebody is discharged, apart from those few areas, the sanctuary sites where infection could still be harbored, somebody can stay with them and not infect them.* – Interview 15
**Individual**	
Lived experiences	*You know, two people can be affected by the same pathogen, but their lived experience is different. And therefore, when we're reading our health statistics, you know, we have decided to classify diseases using pathogens but that in itself is already an impoverishment of data because the lived experiences can be quite radically different. And these are things that often get missing in the so-called evidence based policy making. Because the evidence you're using is already selected and filtered.* – Interview 21
Resilience	*For me as a person, from my point of view, I thought it was just a knowledge gap on their side that they would stigmatise us. Me, I didn't take it seriously in my mind. I work in the ETU and here I am a survivor.* – Interview 27
Agency	*All the models with fear appeals and the general finding across different areas is that fear appeals themselves are not as effective as they could be if they are not accompanied by aspects strengthening the coping responses at the same time to really provide appropriate and easily actionable steps that can be taken that are easy to do.* – Interview 17
